# Analysis of Demineralized Chemical Substances for Disinfecting Gutta-percha Cones

**DOI:** 10.22037/iej.v13i3.18950

**Published:** 2018

**Authors:** George Táccio de Miranda Candeiro, Eduardo Akisue, Fabrícia Campelo Correia, Edmilson dos Santos Sousa, Mônica Sampaio do Vale, Elaine Faga Iglecias, Giulio Gavini

**Affiliations:** a * Department of Restorative Dentistry, Faculty of Dentistry, University of São Paulo, São Paulo, Brazil; *; b * Post Graduation Program in Dental Sciences, Universitary Center Christus, Fortaleza, Brazil; *; c * Discipline of Endodontics, Faculty of Dentistry, University Santa Cecilia, Santos Brazil;*; d * Department of Dental Clinic, Faculty of Pharmaceutics, Dentistry and Nursy, Federal University of Ceará, Fortaleza, Brazil*

**Keywords:** Chemical Substances, Disinfection, Gutta-Percha, Irrigating Solution

## Abstract

**Introduction:**

The aim of the present research was to evaluate the effectiveness of 5% malic acid, 17% EDTA and 10% citric acid solutions used to disinfect gutta-percha cones contaminated by *Enterococcus* *faecalis* (ATCC 29212).

**Methods and Materials:**

Two hundred and ten previously sterilized gutta-percha cones were contaminated with *E. faecalis *at concentration of 1.5×10^8 ^CFU/mL. The cones were immersed in 5% malic acid, 17% EDTA, 10% citric acid, 1% NaOCl and 2.5% NaOCl for 1, 5 and 10 min. Then each cone was kept in Eppendorf tubes containing BHI sterile solution at 37^°^C for 48 h. The presence of turbidity in BHI solution was analyzed. The results were statistically analyzed by Kruskal-Wallis test and 5% Dunn comparisons. *P*-value was considered statistically significant when *P*<0.05.

**Results:**

Regardless of exposure time, 1% NaOCl and 2.5% NaOCl were the most effective agents for rapid disinfection of gutta-percha cones (*P*<0.001). All specimens immersed in experimental demineralized solutions presented bacterial growth (*P*>0.05).

**Conclusion:**

Demineralized solutions tested were not effective for elimination of *Enterococcus faecalis* on the surface of gutta-percha cones.

## Introduction

The importance of rapid disinfection of gutta-percha (GP) cones during endodontic treatment for not breaking the asepsis chain and to prevent bacterial contamination of the root canal is widely recognized in endodontic practice [1]. 

Sodium hypochlorite is the most studied chemical solution for disinfecting gutta-percha cones [2-5]. The antimicrobial activity of NaOCl was related to its concentration and time, *i.e.,* higher concentrations took less time to inhibit bacterial growth than lower concentrations. However, some researches have reported damages on gutta-percha surface after being exposed to sodium hypochlorite in high concentrations [5, 6]. Other chemical substances are also used to promote the fast elimination of microorganisms from gutta-percha cones, such as MTAD [7], chlorhexidine [4, 7], paracetic acid [8, 9] and more recently, herbal oils and extracts [10].

In endodontic therapy, several substances are used to remove smear layer, such as ethylenediaminetetraacetic acid (EDTA) [11], citric acid [12, 13], apple vinegar [14] and malic acid [13]. Currently, there are few studies about the antibacterial effectiveness of these solutions. Moreover, a considerable capacity in eliminating microorganisms have been demonstrated by EDTA and citric acid [11, 12, 15, 16]. However, up to present moment, there are no studies assessing the effectiveness of these substances in promoting rapid disinfection of gutta-percha cones.

The aim of the present study was to evaluate, at different contact times, the effectiveness of 5% malic acid, 17% EDTA and 10% citric acid used to disinfect gutta-percha cones previously contaminated by *Enterococcus (E.) faecalis* (ATCC 29212) and to compare their effectiveness with 1% NaOCl and 2.5% NaOCl. The nulls hypotheses of this study were *i)* the types of chemical substances and *ii)* the exposure times have no influence on the disinfection of gutta-percha cones.

## Materials and Methods

***Specimen preparation***

Two hundred and ten 28 mm, medium (M) size GP cones (Tanari^®^, Manacapuru, AM, Brazil) were randomly divided into five experimental groups and two control groups. The cones were previously sterilized by immersion in 2% glutaraldehyde solution for 10 h. After this period, the cones were rinsed with 50 mL of saline for six min in order to remove any residue of glutaraldehyde from the surface of GP cones. The cones were transferred to sterile filter paper and placed in a dry heat sterilizer for 30 min at 37^°^C until dry.

***Microorganism inoculum preparation***

The bacterial strain used in this experiment were obtained from the American Type Culture Collection (ATCC, Rockville, MD, USA) and maintained frozen at -70^°^C, in 10% skim milk (Difco Laboratories, Detroit, MI, USA), containing 5% of glycerol (Merck, Darmstadt, Germany). *Enterococcus faecalis* strains (ATCC 29212) were cultivated and kept in proper atmosphere and medium. To standardize the bacterial suspension, the samples were diluted and counted to obtain a suspension of approximately 1.5×10^8^ colony forming units per millimeter of suspension (CFU/mL), corresponding to 1 of McFarland scale.

***Specimen contamination***

Gutta-percha cones, medium size, 28 mm, were placed on petri dishes containing bacterial suspension (1.5×10^8^ CFU/mL) of *E. faecalis* and kept immersed at 37^°^C for 72 h. 

***Specimen disinfection***

After contamination, the GP cones were transferred to sterile filter paper and placed in a dry heat sterilizer for 30 min at 37^°^C until dry. The cones were immersed on petri dishes with 10 mL of 5% malic acid, 17% EDTA, 10% citric acid, 1% NaOCl and 2.5% NaOCl for 1, 5 and 10 min. After, GP cones were rinsed with 5% sodium thiosulfate solutions, when immersed in NaOCl. When immersed in other substances, the cones were rinsed with sterile deionized water, to neutralize those substances. In sequence, each cone was transferred to individual Eppendorf tubes containing 2 mL of BHI nutrition medium (Difco Laboratories, Detroit, MI, USA), kept at 37^°^C for 48 h. 

***Analysis procedure***

After incubation, the tubes were analyzed by two observers that confirmed the presence of turbidity in the medium, as an indicator of microbial growth. Agar plates were inoculated with 10 mL from each test tube, and were left at 37^o^C for 24-48 h in appropriate gaseous conditions (as described above) to investigate all possible microbial growth. The purity of the positive cultures was confirmed by gram staining, by colony morphology on blood agar plates, and by the use of biochemical identification kits (API 20 Strep, API CAUX, API 20 Staph, and Rapid ID32A; BioMérieux, Marcy-l’Etoile, France).

***Control groups***

In negative control group (*n*=30), sterilized cones were not contaminated and were placed in Eppendorf tubes containing 2 mL of Brain and Heart Infusion (BHI) medium (Difco Laboratories, Detroit, MI, USA), incubated at 37^°^C for 48 h, in order to evaluate the effectiveness of the sterilization process. In positive control group (*n*=30), contaminated cones were kept immersed in saline on experimental times.

***Scanning electron microscopy (SEM) analysis***

Sixty-five gutta-percha cones were immersed in one of the types of chemical substances (1% NaOCl, 2.5% NaOCl, 10% citric acid, 5% malic acid and 17% EDTA) for 1, 5 or 10 min (*n*=5). Their surfaces were compared with that of a fresh GP cone by scanning electron microscopy (SEM) under ×1000 magnification (JEOL JSEM-820, JEOL Ltd., Tokyo, Japan). The presence of roughness and structural defect were observed and compared with control group in a qualitative analysis.

***Statistical analysis***

The results were statistically analyzed by Kruskal-Wallis test and 5% Dunn comparisons. *P*-value was considered statistically significant when *P*<0.05. All analyses were performed using SPSS 15.0 (SPSS Inc., Chicago, IL, USA). 

**Table 1:** Percent (%) of bacterial growth after direct contact with chemical solutions tested to disinfect gutta-percha cones, according to the exposure time (*P*<0.001)

**Chemical Substance**	**1 min**	**5 min**	**10 min**
**10% Citric Acid**	100.0^Aa^	100.0^Aa^	100.0^Aa^
**5% Malic Acid**	100.0^Aa^	100.0^Aa^	100.0^Aa^
**17% EDTA**	100.0^Aa^	100.0^Aa^	100.0^Aa^
**1% NaOCl**	0.0^Ab^	0.0^Ab^	0.0^Ab^
**2.5% NaOCl**	0.0^Ab^	0.0^Ab^	0.0^Ab^
**Saline**	100.0^Aa^	100.0^Aa^	100.0^Aa^

*Different letters indicate statistically significant difference (P<0.05); Uppercase letters (A and B) and lowercase letters (a and b) are related in the comparison among the exposure times and among the chemicals substances tested, respectively*

**Figure 1:**
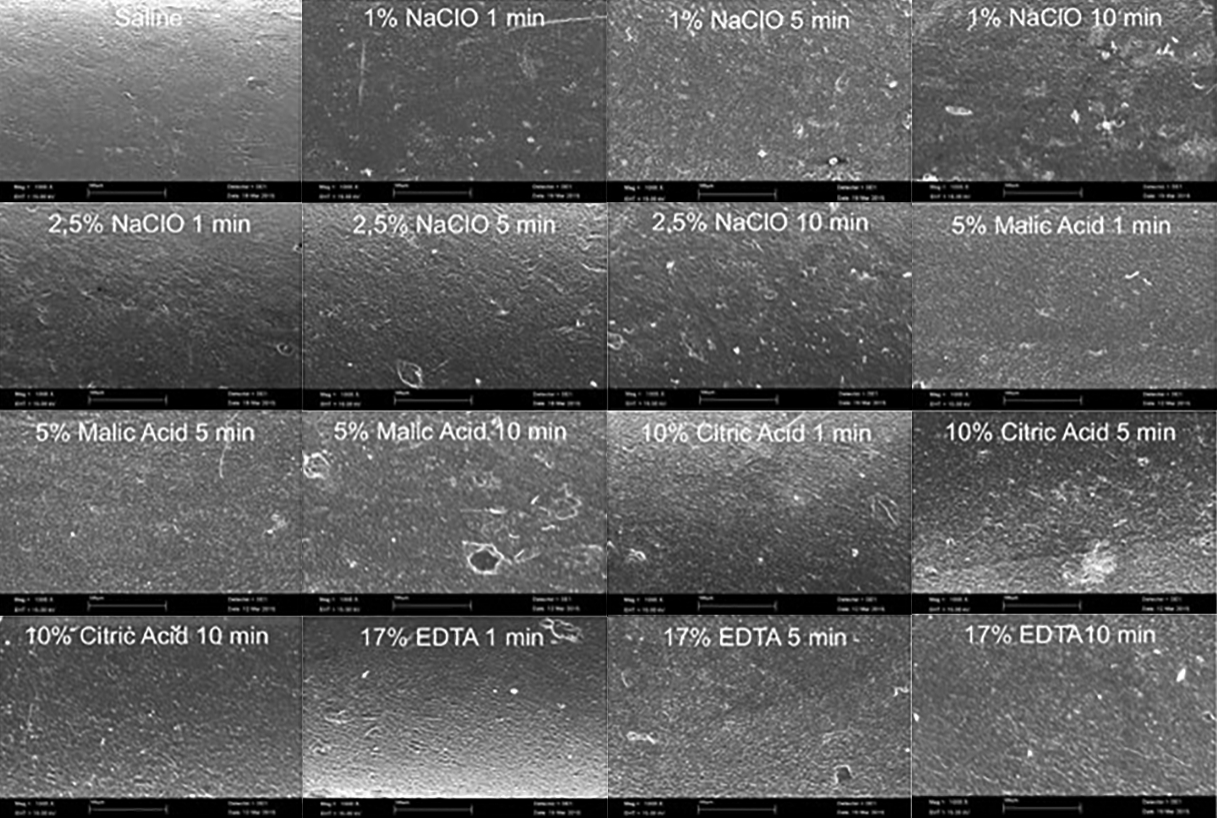
SEM images of the surface of GP cones after 1, 5 and 10 min of contact with each experimental irrigating solutions (magnification ×1000

## Results

The results of all disinfection procedures tested are exposed in Table 1. Regardless of exposure time, 1% NaOCl and 2.5% NaOCl were the most effective agents for rapid disinfection of gutta-percha cones (*P*<0.001). It was observed that in exposure times of 1, 5 and 10 min, 10% citric acid, 5% malic acid and 17% EDTA were unable to eliminate *E. faecalis* in 100% of specimens. 

All positive controls (saline) showed positive results during the analysis and no microbial growth was observed in negative control, confirming the efficacy of previous sterilization.

Analyzing the gutta-percha surfaces, SEM images under ×1000 magnification, when compared 1% and 2.5% sodium hypochlorite solutions to the control group, these solutions promote morphological changes and roughness only after the period of 5 min without qualitative difference between these two concentrations. The same observation was obtained when comparing the control group to the 10% citric acid solution. The other acid solutions (5% Malic acid and 17% EDTA) promoted morphological changes and roughness in all experimental periods of 1, 5 and 10 min.

## Discussion

The aim of the present work was to evaluate the effectiveness of demineralized chemical substances to disinfect gutta-percha cones contaminated previously with *E. faecalis*. The null hypothesis was accepted in relation to chemical substances tested but it was rejected in relation to exposure time.

The present study evaluated, in gutta-percha cones previously infected with *E. faecalis,* the antibacterial effectiveness of demineralized solutions applied to remove smear layer. According to methodology performed, 5% malic acid, 10% citric acid and 17% EDTA exhibited no capacity to disinfect gutta-percha cones contaminated with *E. faecalis.*

Previous researches showed that the bacterial biofilm might grow on gutta-percha surface [17, 18]. Contamination of gutta-percha cones may occur by handling, aerosols, and also by physical sources during the storage process, which gives no sterility guarantee [3-5]. In clinical practice, a rapid chemical disinfection is needed before the introduction of gutta-percha cones in the root canal [9, 18]. Therefore, there is a continuous need for the discovery and development of new antimicrobial therapeutic agents, which might improve the disinfection of endodontic materials, once that the maintenance of the aseptic chain is an essential factor for the success in endodontic therapy [1, 8, 9].

EDTA is a calcium and magnesium chelator and it is the most applied solution to remove smear layer after root canal instrumentation [11, 12]. EDTA still presents an inhibitory activity against gram-negative bacteria, *staphylococci* and *Candida (C.) spp *[19-21]. While its activity against gram-negative bacteria was demonstrated to be because of metal ions chelation [21], its mode of action against *staphylococci* is not clear [18]. Its activity against *C. albicans* was demonstrated to be because of yeast-to-mycelium transition blockage [20]. Recent publication indicated that EDTA alone and in combination is an effective antibiofilm agent with a spectrum covering both gram-positive and gram-negative bacteria as well as *C. albicans *[21]*. *Its antibiofilm effect was suggested to be because of its capability to sequester the divalent ions essential to the extracellular polymeric matrix structure of biofilms [16, 21] that result in detaching bacteria from this structure [22]. Although the mode of action of EDTA in this respect is still not completely clarified. Perhaps, the chelating effect of calcium (Ca^2+^), magnesium (Mg^2+^) and iron (Fe^2+^) ions may affect important metabolic pathways in the bacterial cell [17]. It was observed that EDTA decreased significantly *P. aeruginosa, E. coli* and *C. albicans* only after 4 h of direct contact [21]. However, no bactericidal effect against *E. faecalis *was observed using a 17% EDTA solution, even after 60 min of contact time [12, 22]. The present study is in agreement with Arias-Moliz *et al*. [15] and de Almeida *et al. *[22], who reported that 17% EDTA solution showed no bactericidal activity against *E. faecalis.*

Some other substances have been proposed to remove smear layer, as an alternative to EDTA, such as citric acid and malic acid [13]. These substances present favorable biological properties and they are able to remove smear layer [13, 23]. In relation of its antibacterial activity applied in Endodontics, there still are few studies. Some authors reported that 10% citric acid had activity against *E. faecalis* after 10 min in contact with bovine dentin [12,15], but these results cannot be compared with others due to the difference of methodologies. The alternated use of citric acid and 1% sodium hypochlorite presented greater antibacterial effectiveness against *E. faecalis* and *C. albicans* when compared with 1% sodium hypochlorite alone during root canal preparation [24].

Malic acid (molecular weight=134.09 Daltons) is an organic acid that may be transformed in maleic acid (molecular weight=116.03 Daltons) if lost a water molecule (desiderated reaction); and according to previous study this molecule can attain a significant percentage of inhibition of *E. faecalis* biofilm formation and present a high antimicrobial capacity [13]. Some authors have reported that the antimicrobial activity might reside in the chemical nature of organic acids, mainly by its very acidic pH that decrease the internal pH of the microbial cell and alters cell membrane permeability [19] and maintain a great residual activity [24]. However, in the present research, the low pH of citric acid and malic acid (1.76 and 2.02, respectively) had no influence in elimination of *E. faecalis*. 

Sodium hypochlorite, in several concentrations, is the most widely substance used in endodontic therapy and the most studied chemical solution to disinfecting gutta-percha cones [11]. The present research showed that 1% and 2.5% sodium hypochlorite presented high capacity to eliminate *Enterococcus faecalis* from gutta-percha cones even when exposure time was 1 min. Our results agree with the findings of previous studies [2, 3], regarding the time taken by lower concentrations of NaOCl to exert antimicrobial activity. On the other hand, higher concentrations showed that 1 min immersion time of gutta-percha cones in 5.25% sodium hypochlorite accomplished sterilization against a variety of gram-positive, gram-negative, and spore-forming microorganisms. These results are in agreement with previous studies [7, 25], but most of them evaluated the effectiveness of sodium hypochlorite with exposure times from 5 to 30 min. The present research showed that these substances presented high capacity to eliminate *E. faecalis* from gutta-percha cones. Although Gomes *et al. *[4] reported that 1% and 2.5% NaOCl were effective only in exposure times above of 20 and 10 min, respectively; the present study showed that 1% and 2.5% sodium hypochlorite presented high capacity in elimination *E. faecalis* from gutta-percha cones even when exposure time was 1 min. 

According to Pang *et al.* [6], for root canal filling, a change in the physical properties of GP cones may affect the root canal filling outcome. Deep irregularities, formed through deterioration of GP cones, can create large interfacial gaps between the GP cones and the root canal wall, increasing the risk of leakage. Recently, Brito *et al.* [5] reported that the damages on gutta-percha cones promoted by long periods of immersion in sodium hypochlorite solutions had no influence on microleakage. By the results from the present study, although an exposure time of 1 min either with 1% NaOCl or 2.5% NaOCl was enough to eliminate *Enterococcus faecalis* from gutta-percha cones. SEM analysis showed that the superficial damage was dependent-time in regarding to sodium hypochlorite solutions and 10% citric acid solution. A short time of exposure for gutta-percha disinfection could decrease the risk of damages on the gutta-percha surface, once that high exposure time may promote alteration in their surface morphology, mainly roughness. Other solutions as 5% malic acid and 17% EDTA promoted alterations in the surface of gutta-percha cones in any exposure time. Aktemur Turker *et al.* [8] using SEM/EDS analyses observed no significant alterations in gutta-percha cones when immersed in sodium hypochlorite for 5 and 10 min.

## Conclusion

Within the limitations of the present study, these results showed that, regardless of exposure time, 5% malic acid, 10% citric acid and 17% EDTA were not effective to eliminate *Enterococcus faecalis* on surface of gutta-percha cones. It was also observed that 1% NaOCl and 2.5% NaOCl were effective agents for the rapid disinfection of gutta-percha cones before root canal filling.
